# *De novo* intronic *GATA1* mutation leads to diamond-blackfan anemia like disease

**DOI:** 10.3389/fgene.2023.1068923

**Published:** 2023-02-10

**Authors:** Shan Liu, Kunlin Pei, Lu Chen, Jing Wu, Qiuling Chen, Jinyan Zhang, Hui Zhang, Chengyi Wang

**Affiliations:** ^1^ Department of Hematology & Oncology, Fujian Children’s Hospital, Fujian Branch of Shanghai Children’s Medical Center Affiliated to Shanghai Jiaotong University School of Medicine, Fuzhou, China; ^2^ Shanghai Children’s Medical Center, Shanghai Jiao Tong University School of Medicine, Shanghai, China; ^3^ Department of Hematology/Oncology, Guangzhou Women and Children’s Medical Center, Guangzhou Medical University, Guangzhou, China; ^4^ Fujian Medical University, Fuzhou, China

**Keywords:** *de novo* mutation, diamond-blackfan anemia, GATA1, intronic mutation, inherited

## Abstract

*GATA1* is required for normal erythropoiesis. Exonic/intronic *GATA1* mutations causes Diamond-Blackfan Anemia (DBA)-like disease. Herein, we present a case of a 5-year-old boy with anemia of unknown etiology. Whole-exome sequencing revealed a *de novo GATA1* c.220 + 1G>C mutation. The reporter gene assay revealed that such mutations did not affect on GATA1 transcriptional activity. The normal transcription of *GATA1* was disturbed, as evidenced by increased expression of the shorter *GATA1* isoform. RDDS prediction analysis revealed that abnormal *GATA1* splicing might be the underlying mechanism disrupting *GATA1* transcription, thereby impairing erythropoiesis. Prednisone treatment significantly improved erythropoiesis, evidenced by increased hemoglobin and reticulocyte counts.

## Introduction

Diamond-Blackfan Anemia (DBA), a type of congenital bone marrow failure syndrome, is characterized by erythroid aplasia, which is usually accompanied by developmental malformations and malignancy susceptibility (Da Costa et al., 2020). DBA is believed to be a ribosomopathy caused by inherited genetic mutations that interfere with ribosome synthesis. In this regard, ribosomal proteins (RP) (RPS19, RPL5, RPS26, RPL11, RPL35A, RPS10, RPS24, RPS17, RPL15, RPS28, RPS29, RPS7, RPS15, RPS27A, RPS27, RPL9, RPL18, RPL26, RPL27, and RPL31) and RPS26 chaperon (TSR2) are the most affected (Ulirsch et al., 2018). Other non-ribosomopathy DBA have recently been reported and classified as DBA-like syndrome (DBS) (Da Costa et al., 2020).

More than 90% of children with DBA are diagnosed within the first year, and delayed diagnosis rarely occurs (Vlachos et al., 2008). To date, families with DBS or dyserythropoietic anemia with inherited *GATA1* variants have been reported. Of these seven instances, five were *GATA1* coding mutations, while two were non-coding mutations (Sankaran et al., 2012; Klar et al., 2014; Ludwig et al., 2014; Parrella et al., 2014; Abdulhay et al., 2019; van Dooijeweert et al., 2022). *GATA1* c.220 + 1G>C mutation has been reported in transient abnormal hematopoiesis (TAM) in patients with Down syndrome. This mutation causes splicing errors, generating the short form of *GATA1* (GATA1s) (Roberts et al., 2013). Mechanistic studies have reported that the inability to occupy erythroid-specific gene regulatory elements may cause GATA1s′ dyserythropoiesis in DBS (Chlon et al., 2015). Abdulhay et al. demonstrated that intronic mutations might impair *GATA1* splicing (Abdulhay et al., 2019). To date, the role of *GATA1* non-coding mutation has received little attention. *De novo* mutations are the most severe type of uncommon genetic variation, and are typically more harmful than inherited variations.

We report a case of a 5-year-old boy with delayed DBS diagnosis, characterized by macrocytic erythropoietic aplasia, *de novo GATA1* non-coding mutations, and prednisone responsiveness.

## Materials and methods

### Patient samples and cell preparation

The study protocol was approved by the Humanities and Ethics Committee of Fujian Children’s Hospital, Fujian Branch of Shanghai Children’s Medical Center Affiliated to Shanghai Jiaotong University School of Medicine (2022ETKLR07003). The parents and five healthy volunteers provided written informed consent before peripheral blood samples were collected in accordance with institutional policies and the Declaration of Helsinki.

### Cell lines

The Shanghai Children’s Medical Center maintained Ccryopreserved K562 and 293T cells. The 293T cells were maintained in DMEM supplemented with 10% fetal bovine serum (FBS) (Sigma), and the K562 cells were maintained in RPMI 1640 medium supplemented with 10% FBS. All the cells were grown in a 37°C incubator with 5% CO_2_.

### Whole-exome sequencing and bio-informatic analysis

Genomic DNA was isolated from the patient’s and his parent’s peripheral blood, while an oral scraping sample was collected as a germline control. Whole-exome sequencing (WES) was performed by inputting 500 ng of genomic DNA from the patient. Briefly, the WES library was prepared by KindStar, Inc. Exome capture using the MGIEasy Exome Capture V4 Probe (MGI) was followed by paired-end read sequencing (2 × 100 bp read length) on the MGISEQ-2000 platform, with an average depth of ≥100-fold. Exome sequencing data were analyzed as described previously (Ulirsch et al., 2018).

### Sanger sequencing

The *GATA1* genotype of samples from the patient’s blood and oral scrapings, and parents’ blood was examined using Sanger sequencing. Polymerase chain reaction (PCR) was performed to amplify the relevant DNA fragments using the primers specified in Supplementary Table S1.

### *GATA1* intronic enhancer activity

A 320-bp region encompassing GATA1 ChrX: 48,649,737 was amplified using CloneAmp HiFi PCR Premix (Clontech) (primer sequences listed in Supplementary Table S1) and cloned into the pGL4.23-mini/P vector with a minimal SV40 promoter upstream of the firefly luciferase gene sequence. Mutagenesis was performed using the QuikChange II Site-Directed Mutagenesis Kit (Agilent Technologies), with the primers listed in Supplementary Table S1. For reporter assays, 2 × 10^6^ K562 cells were resuspended in 100 μL of Nucleofector Solution Kit V (Lonza) with the addition of 1.9 μg of pGL4.23 constructs and 100 ng of renilla pTK plasmid. The cells were electroporated and incubated for 24 h at 37°C with 5% CO_2_. Similarly, HEK293T cells (6 × 10^4^) were plated in 96-well plates (flat bottom), co-transduced with 95 ng pGL4.23 constructs and 5 ng renilla pTK, and incubated for 24 h. The experiments were performed in triplicate. Firefly luciferase activity was normalized to the Renilla luciferase activity to control for cell number and transfection efficiency. Measurements are presented as a ratio relative to the activity of the pGL4.23-mini/P vector with the wild-type G allele.

### Real-time quantitative PCR

Total RNA was extracted using the RNeasy Micro Kit (Qiagen, Hilden, Germany) according to the manufacturer’s protocol. We reverse transcribed 500 ng of RNA into cDNA, and the qRT-PCR was performed using an ABI Prism 7900HT detection system (Applied Biosystems) with FastStart SYBR Green master mix (Roche). *GAPDH* was used as an internal control. The primer sequences used in this study are listed in Supplementary Table S1.

### Flow cytometry

EDTA-treated blood was stained with monoclonal PE-conjugated CD33, PE-conjugated CD55, and FITC-conjugated CD59 and analyzed by flow cytometry as previously described (Abdulhay et al., 2019).

### Chromosomal microarray assay

Genomic DNA was isolated using the QIAamp DNA Blood Mini Kit (Qiagen, Hilden, Germany). A chromosomal microarray assay was performed using a high-resolution genotyping single nucleotide polymorphism microarray (Affymetrix CytoScan 750 K Array; Affymetrix, Santa Clara, CA, United States). CNVs were identified based on records associated with the human reference genome 37 (hg19) of the National Center for Biotechnology Information.

### Statistical analysis

SPSS version 26.0 for Windows (Armonk, NY, IBM Corp) was used for data analysis. *p* < 0.05 was considered to be statistically significant.

## Results

### Case presentation

**FIGURE 1:**
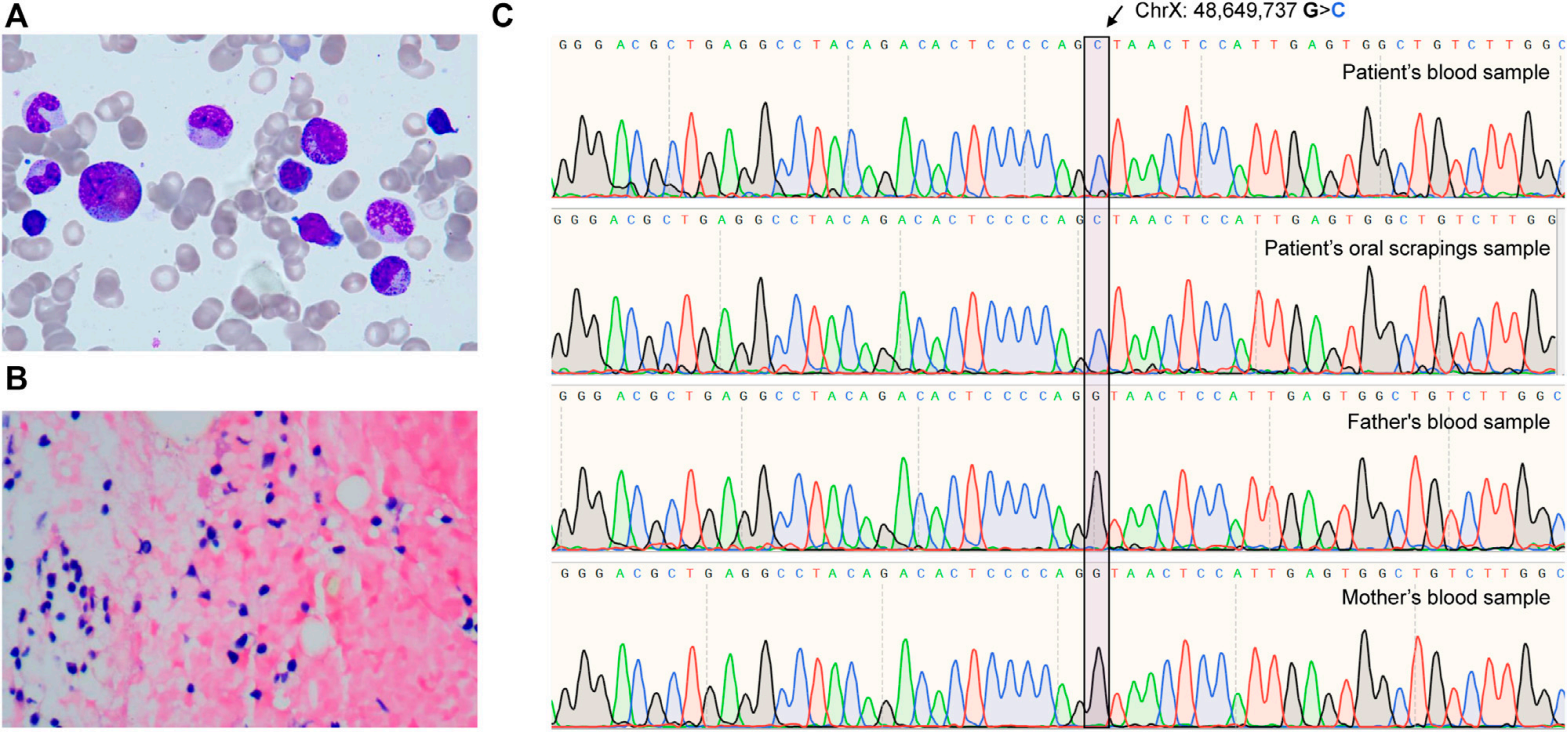
The *de novo GATA1* mutation-induced Diamond-Blackfan-like syndrome. **(A)** Images of bone marrow aspirates from patients taken with 100 × magnification. **(B)** A 40 × magnification bone marrow sample revealed hypercellular bone marrow and a florid population with reduced erythropoiesis. **(C)** Sequencing chromatograms from patient’s and parents’ *GATA1* mutations (chrX: 48,649,737 G>C in hg19).

A 5-year-old child was diagnosed with severe anemia for over 2 years ago. Physical examination revealed pallor without obvious deformities, palpable lymphadenopathy, or hepatosplenomegaly. Laboratory tests revealed mild macrocytic anemia with 94.5–99.6 fL mean corpuscular volume, as indicated by a 44 g/L hemoglobin level and an absolute reticulocyte count of 13.6 × 10^9^/L. Bone marrow aspiration (BMA) revealed erythroid aplasia with normal granulopoiesis and thrombocytopoiesis (Figures 1A, B). Autoimmune and infectious diseases were excluded from systemic laboratory tests. Therefore, the pure red cell aplasia (PRCA) diagnosis was made. A series of systemic hematopoietic tests were performed to determine the cause further. A bone marrow biopsy revealed 30% bone marrow cellularity and increased adipose tissue proliferation. Abnormal localization of immature precursor cells (ALIP) has not yet been identified. The results of CD55, CD59, and FLAER assays evaluated by flow cytometry were normal (Supplementary Figures S1A, B). No copy number variation was identified among chromosomes 5 and 7 by chromosomal microarray assay (Supplementary Figures S2A, B), suggesting that the possibility of myelodysplastic syndromes (MDS) and paroxysmal nocturnal hemoglobinuria (PNH) was excluded. Whole-exome sequencing (WES) was performed to investigate the genetic alterations underlying this PRCA case. The *GATA1* c.220 + 1G>C mutation found by WES was further confirmed (Figure 1C) by Sanger sequencing, which was not identified in the Genome Aggregation Database (gnomAD) SV database (125,748 genomes) or the 15,708 Genomes database (Karczewski et al., 2020). We performed Sanger sequencing of the patient’s oral scraping sample to confirm the origin of this mutation. Therefore, a *GATA1* germline mutation was identified (Figure 1C). Sanger sequencing of his parents’ blood samples revealed that the *GATA1* c.220 + 1G>C mutation was a *de novo* mutation rather than an inherited variation (Figure 1C). A diagnosis of DBS with non-RP gene mutations was made.

### *De novo GATA1* mutation dysregulated *GATA1* transcription

**FIGURE 2:**
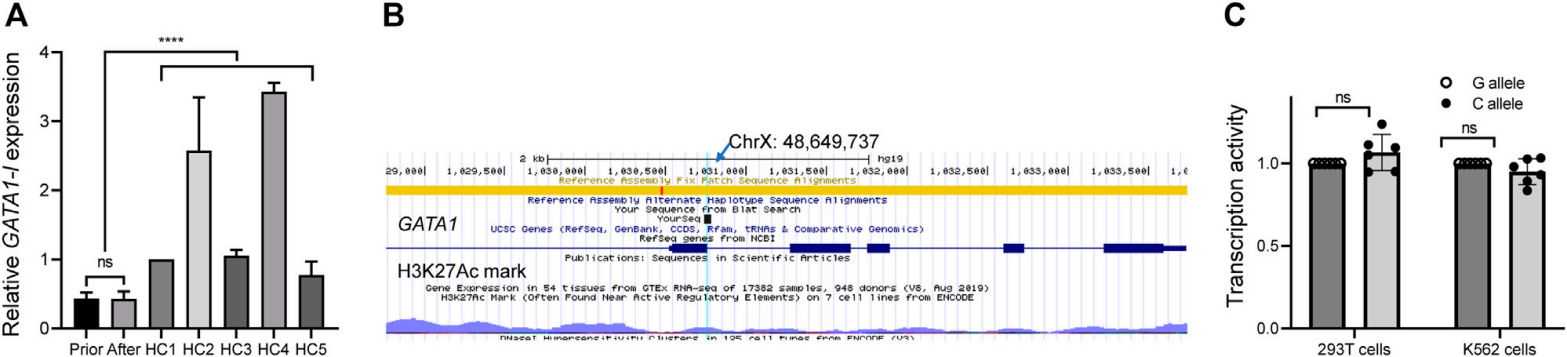
Functional assay of *de novo GATA1* mutation. **(A)** mRNA level of long *GATA1* isoform in peripheral blood mononuclear cells from the patient before and after therapy, as well as from healthy control subjects (HC), ****p < 0.0001. **(B)** Reference nucleotide from the epigenome browser. The arrow shows the point of mutation according to the sequence result. **(C)** A luciferase reporter assay showed that *GATA1* **(C)**220 + 1G>C mutation did not impair the transcriptional activity in 293T cells and K562 cells. Data represent the mean ± standard deviation (SD), ns, no significance.

We performed a qPCR assay to examine *GATA1* transcription to confirm the pathological role of this *de novo* mutation and found that the normal *GATA1* (*GATA1-l*) transcription was significantly suppressed in comparison to healthy controls (N = 5) (*p* < 0.0001) (Figure 2A), indicating the pathogenic role of the mutation. By examining the chromatin state annotations of this genomic region across 42 cells and tissues from the Roadmap Epigenomics Project (Moore et al., 2020), we observed that *GATA1* c.220 + 1G>C mutation was aligned with a putative weak enhancer with H3K27ac enrichment (Figure 2B). To probe the enhancer function of this regulatory DNA element and investigate how its activity is influenced by *GATA1* c.220 + 1G>C, we first tested the 320-bp fragment surrounding *GATA1* c.220 + 1G using a reporter gene assay in 293T cells. The fragment identical to the mutant C allele revealed a transcription effect similar to that of the wild-type allele (Figure 2C). We performed the same reporter assay using K562 cells to exclude the impact of the cellular matrix, an erythroid cell line. Figure 2C shows that *GATA1* c.220 + 1G>C mutation did not impair transcription activity in this erythroid context.

**FIGURE 3:**
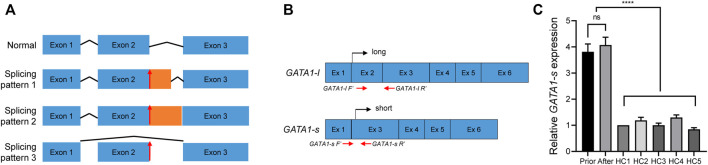
Validation of GATA1 splicing. **(A)** RDDS predicted an abnormal splicing model after *GATA1* **(C)**220 + 1G>C mutation. Lines indicate introns, and blue boxes indicate normal exons, respectively. Orange boxes indicate aberrant exons. Red arrows showed the mutation site. **(B)** Primer design and transcription diagram of long- and short- *GATA1* form. **(C)** mRNA level of short *GATA1* isoform in peripheral blood mononuclear cells from the patient before and after therapy, as well as from healthy control subjects (HC), ****p < 0.0001.

Because such intronic mutations did not impair *GATA1* transcriptional activity (Figures 2B, C), we hypothesized that they might alter the splicing process. To preliminarily probe this possibility, we used the RNA Splicer analytical tool developed by the Research Institute of Tsinghua (https://rddc.tsinghua-gd.org/). *GATA1* c.220 + 1G>C mutation potentially disturbed the *GATA1* normal transcription *via* three alternative splicing models (Figure 3A). We designed two primer pairs to determine the splicing effect on *GATA1* transcription and quantify the two *GATA1* isoforms (Figure 3B). The *GATA1* short isoform (*GATA1-s*) was significantly higher than that in healthy controls (N = 5) (*p* < 0.0001) (Figure 3C). We found that the *GATA1* short isoform was expressed preferentially after relating these data to the readings of the *GATA1* long isoform (Figures 2A, 3C).

### Glucocortisteroid treatment response

**FIGURE 4:**
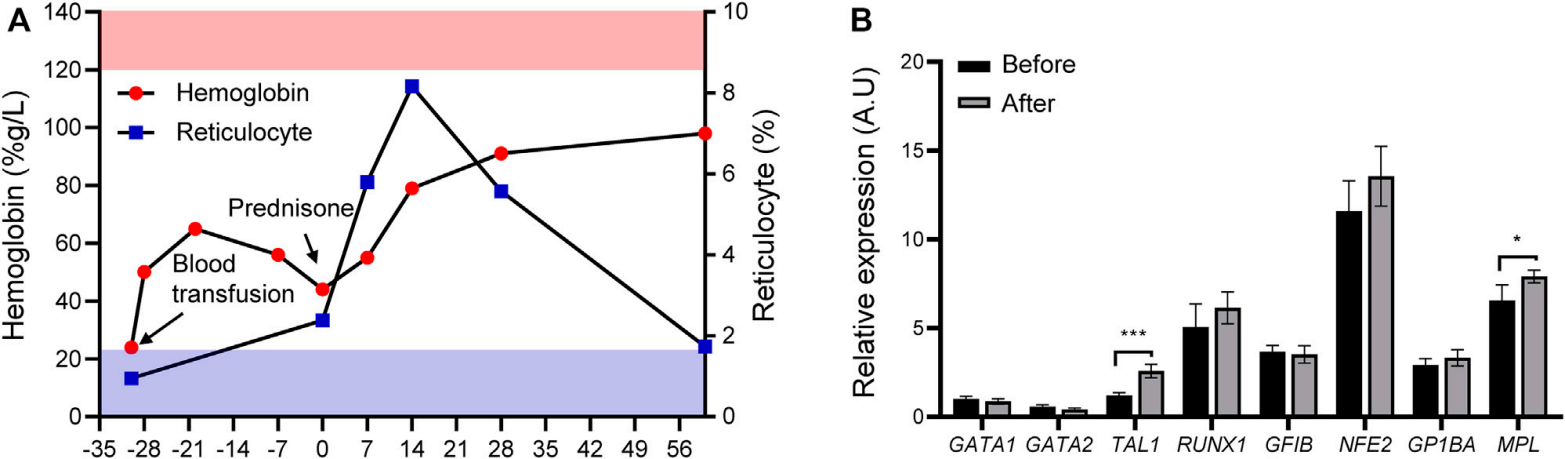
Treatment response and possible transcription reprogramming. **(A)** Treatment response after prednisone (upper panel, pink stripes, normal range of hemoglobin; lower panel, blue stripes, normal range of reticulocyte; solid red circle, hemoglobin; solid blue square, reticulocyte) and treatment schema (Black arrows, blood transfusion and prednisone). **(B)** Expression of erythroid developmental target genes (*TAL1*, *MPL*, *GATA2*, *RUNX1*, *GFIB*, *GP1BA*, and *NFE2*) before and after prednisone treatment. *p < 0.05, ***p < 0.001, ****p < 0.0001.

Before this diagnosis, red blood cell transfusion was occasionally prescribed to relieve the symptoms. Building upon DBS diagnosis, we treated the patient with prednisone at a dose of 2 mg/kg/d, which was tampered with after 2 weeks of treatment. We evaluated the treatment response through routine total cell counts and reticulocyte percentage measurements over the first month. Figure 4A shows that the hemoglobin level gradually increased from 44 to 91 g/L, accompanied by a concurrent reticulocyte increase, indicating a response effect. After 2 months of therapy, the hemoglobin level remained steady over the follow-up period, between 91 and 98 g/L (Figure 4A). We performed qPCR to investigate the effect of prednisone on *GATA1* transcription and evaluate *GATA1* transcription alteration after prednisone exposure. Figures 2A, 3C, 4B show that either *GATA1-l* or *GATA1-s* transcription was affected by prednisone treatment, further confirming the splicing error due to this *de novo* intronic mutation. We selected several erythropoiesis-related genes to primarily investigate the glucocorticosteroid responding program and perform the qPCR assay. We observed that *TAL1* and *MPL* were up-regulated in the assay, while other genes (*GATA2*, *RUNX1*, *GFIB*, *GP1BA*, and *NFE2*) were not altered (Figure 4B), suggesting a treatment-induced transcriptional reprogramming induced by glucocortisteroids.

## Discussion

We report for the first time a case of DBS caused by a spontaneous mutation in the intron of *GATA1* c.220 + 1G>C, which belongs to the classical splicing site, resulting in a decrease in *GATA1* mRNA transcription (Figure 1). According to our results, the etiology of DBS may be aided by *de novo* GATA1 c.220 + 1G>C impairment of GATA1 splicing. To the best of our knowledge, DBA-like syndrome is associated with this unique *GATA1* mutation.

Most patients with DBA are diagnosed within the first year of life, and this patient was diagnosed with DBS at the age of 5 years. DBA/DBS is a heterogeneous disease with an inherited disorder characterized by abnormal hematopoiesis, congenital malformations, and endocrine dysfunction. Therefore, DBS manifestation linked to GATA1 is comparable to, but not the same as, DBA produced by the traditional RP gene mutation. In addition to macrocytic anemia, this patient showed moderate neutropenia, and aberrant megakaryocyte thrombopoiesis, similar to GATA1-linked DBS. However, no developmental abnormalities were seen in his case (Iskander et al., 2019; van Dooijeweert et al., 2022). DBS caused by GATA1 deficiency has different phenotypic characteristics, including abnormal erythropoiesis and megakaryocytes, and neutropenia. Therefore, it represents different disease phenotypes in DBS spectrum and must be carefully addressed. Panel-captured exome sequencing was performed at another general hospital to focus on myelodysplastic syndromes, which is another reason for the delayed diagnosis. Thus, a thorough understanding of pediatric hematology and oncology is essential because the causes of anemia differ depending on the patient’s age at presentation, sex, race, and ethnicity.

*GATA1* is highly expressed during erythrocyte commitment, especially at the colony-forming unit (CFU-E) stage. At this stage, *GATA1* downregulation potentiates CFU-E apoptosis, thus participating in DBS pathogenesis (Ribeil et al., 2007). GATA1 translation disruption confers erythropoiesis aplasia (Boultwood and Pellagatti, 2014). Notably, a *GATA1* transcriptional decrease was observed in this patient. Evidence has demonstrated that *GATA1* intronic mutations can lead to DBA-like disease *via* an abnormal splicing mechanism. The RDDS prediction tool demonstrated that *GATA1* c.220 + 1G>C mutation produced an important new splice acceptor site. Initially, the sequence at the beginning of intron 2 was “GTAACT”, which mutated to “CTAACT”. The “GT-AG” rule states that novel splicing sites may be generated before the proper splicing site, disrupting GATA1 transcription and compromising protein function (van Dooijeweert et al., 2022). Previous studies have suggested that 9%–30% causative variants in Mendelian disorders may act through splicing disruption (Stenson et al., 2017). However, a “GT-AG” rule cannot be applied to all. Thus, further experimental validation is highly needed to illuminate the aberrant splicing patterns.

This patient received 2 mg/kg prednisone daily for 2 weeks before being tampered with, and a favorable response to therapy was shown. His hemoglobin and reticulocyte levels gradually increased during the first month of treatment. Currently, the response mechanism of *GATA1* mutation-induced DBS to glucocorticoid treatment includes 1) induction of burst- forming unit-erythroid (BFU-E) cell proliferation *via* stress erythropoiesis (Iskander et al., 2021), 2) regulation of P53 signaling (Wang et al., 2022), 3) deactivation of c-Myc (Boultwood and Pellagatti, 2014) and 4) inhibition of mTOR signaling (Payne et al., 2012). In this regard, samples before and after prednisone exposure were collected for testing. Notably, prednisone therapy did not affect on *GATA1* transcription, suggesting a covert mechanism for the positive response to treatment. Although glucocorticoids have been proven to improve erythropoietic recovery in children with GATA1 mutation-induced DBS, the exact mechanism by which glucocorticoids aid in this recovery remains unknown.

## Conclusion

A *de novo GATA1* c.220 + 1G>C mutation was discovered in a 5-year-old boy with chronic anemia. Preliminary mechanistic studies have revealed that this spontaneous intronic mutation has a negative impact on erythropoiesis and *GATA1* transcription. Prednisolone treatment improved anemia quickly. Further research is needed to determine the true molecular mechanism of intronic *GATA1* mutation-induced DBA-like syndrome and how prednisone restores normal erythropoiesis.

## Data Availability

The data presented in the study are deposited in the https://bigd.big.ac.cn/gsa-human/browse/HRA002675 repository, accession number HRA001017.

## References

[B1] Abdulhay N. J. Fiorini C. Verboon J. M. Ludwig L. S. Ulirsch J. C. Zieger B. (2019). Impaired human hematopoiesis due to a cryptic intronic GATA1 splicing mutation. J. Exp. Med. 216 (5), 1050–1060. 10.1084/jem.20181625 30914438 PMC6504223

[B2] Boultwood J. Pellagatti A. (2014). Reduced translation of GATA1 in Diamond-Blackfan anemia. Nat. Med. 20 (7), 703–704. 10.1038/nm.3630 24999938

[B3] Chlon T. M. McNulty M. Goldenson B. Rosinski A. Crispino J. D. (2015). Global transcriptome and chromatin occupancy analysis reveal the short isoform of GATA1 is deficient for erythroid specification and gene expression. Haematologica 100 (5), 575–584. 10.3324/haematol.2014.112714 25682601 PMC4420206

[B4] Da Costa L. Leblanc T. Mohandas N. (2020). Diamond-Blackfan anemia. Blood 136 (11), 1262–1273. 10.1182/blood.2019000947 32702755 PMC7483438

[B5] Iskander D. Roberts I. Rees C. Szydlo R. Alikian M. Neale M. (2019). Impaired cellular and humoral immunity is a feature of Diamond-Blackfan anaemia; experience of 107 unselected cases in the United Kingdom. Br. J. Haematol. 186 (2), 321–326. 10.1111/bjh.15915 30980390

[B6] Iskander D. Wang G. Heuston E. F. Christodoulidou C. Psaila B. Ponnusamy K. (2021). Single-cell profiling of human bone marrow progenitors reveals mechanisms of failing erythropoiesis in Diamond-Blackfan anemia. Sci. Transl. Med. 13 (610), eabf0113. 10.1126/scitranslmed.abf0113 34516827

[B7] Karczewski K. J. Francioli L. C. Tiao G. Cummings B. B. Alfoldi J. Wang Q. (2020). The mutational constraint spectrum quantified from variation in 141,456 humans. Nature 581 (7809), 434–443. 10.1038/s41586-020-2308-7 32461654 PMC7334197

[B8] Klar J. Khalfallah A. Arzoo P. S. Gazda H. T. Dahl N. (2014). Recurrent GATA1 mutations in Diamond-Blackfan anaemia. Br. J. Haematol. 166 (6), 949–951. 10.1111/bjh.12919 24766296

[B9] Ludwig L. S. Gazda H. T. Eng J. C. Eichhorn S. W. Thiru P. Ghazvinian R. (2014). Altered translation of GATA1 in Diamond-Blackfan anemia. Nat. Med. 20 (7), 748–753. 10.1038/nm.3557 24952648 PMC4087046

[B10] Moore J. E. Purcaro M. J. Pratt H. E. Epstein C. B. Shoresh N. Adrian J. (2020). Expanded encyclopaedias of DNA elements in the human and mouse genomes. Nature 583 (7818), 699–710. 10.1038/s41586-020-2493-4 32728249 PMC7410828

[B11] Parrella S. Aspesi A. Quarello P. Garelli E. Pavesi E. Carando A. (2014). Loss of GATA-1 full length as a cause of Diamond-Blackfan anemia phenotype. Pediatr. blood cancer 61 (7), 1319–1321. 10.1002/pbc.24944 24453067 PMC4684094

[B12] Payne E. M. Virgilio M. Narla A. Sun H. Levine M. Paw B. H. (2012). L-Leucine improves the anemia and developmental defects associated with Diamond-Blackfan anemia and del(5q) MDS by activating the mTOR pathway. Blood 120 (11), 2214–2224. 10.1182/blood-2011-10-382986 22734070 PMC3447780

[B13] Ribeil J. A. Zermati Y. Vandekerckhove J. Cathelin S. Kersual J. Dussiot M. (2007). Hsp70 regulates erythropoiesis by preventing caspase-3-mediated cleavage of GATA-1. Nature 445 (7123), 102–105. 10.1038/nature05378 17167422

[B14] Roberts I. Alford K. Hall G. Juban G. Richmond H. Norton A. (2013). GATA1-mutant clones are frequent and often unsuspected in babies with Down syndrome: Identification of a population at risk of leukemia. Blood 122 (24), 3908–3917. 10.1182/blood-2013-07-515148 24021668 PMC3995281

[B15] Sankaran V. G. Ghazvinian R. Do R. Thiru P. Vergilio J. A. Beggs A. H. (2012). Exome sequencing identifies GATA1 mutations resulting in Diamond-Blackfan anemia. J. Clin. investigation 122 (7), 2439–2443. 10.1172/JCI63597 PMC3386831 22706301

[B16] Stenson P. D. Mort M. Ball E. V. Evans K. Hayden M. Heywood S. (2017). The human gene mutation database: Towards a comprehensive repository of inherited mutation data for medical research, genetic diagnosis and next-generation sequencing studies. Hum. Genet. 136 (6), 665–677. 10.1007/s00439-017-1779-6 28349240 PMC5429360

[B17] Ulirsch J. C. Verboon J. M. Kazerounian S. Guo M. H. Yuan D. Ludwig L. S. (2018). The genetic landscape of diamond-blackfan anemia. Am. J. Hum. Genet. 103 (6), 930–947. 10.1016/j.ajhg.2018.10.027 30503522 PMC6288280

[B18] van Dooijeweert B. Kia S. K. Dahl N. Fenneteau O. Leguit R. Nieuwenhuis E. (2022). GATA-1 defects in diamond-blackfan anemia: Phenotypic characterization points to a specific subset of disease. Genes 13 (3), 447. 10.3390/genes13030447 35328001 PMC8949872

[B19] Vlachos A. Ball S. Dahl N. Alter B. P. Sheth S. Ramenghi U. (2008). Diagnosing and treating Diamond blackfan anaemia: Results of an international clinical consensus conference. Br. J. Haematol. 142 (6), 859–876. 10.1111/j.1365-2141.2008.07269.x 18671700 PMC2654478

[B20] Wang B. Wang C. Wan Y. Gao J. Ma Y. Zhang Y. (2022). Decoding the pathogenesis of Diamond-Blackfan anemia using single-cell RNA-seq. Cell Discov. 8 (1), 41. 10.1038/s41421-022-00389-z 35534476 PMC9085895

