# Supplementary material for: De novo intronic GATA1 mutation leads to diamond-blackfan anemia like disease
Source: Front Genet. 2023 Feb 10;14:1068923. doi: 10.3389/fgene.2023.1068923 (PMC9950261; doi:10.3389/fgene.2023.1068923)
Supplement: Supplementary file 1 [file Presentation1.PPTX]

## Slide 1
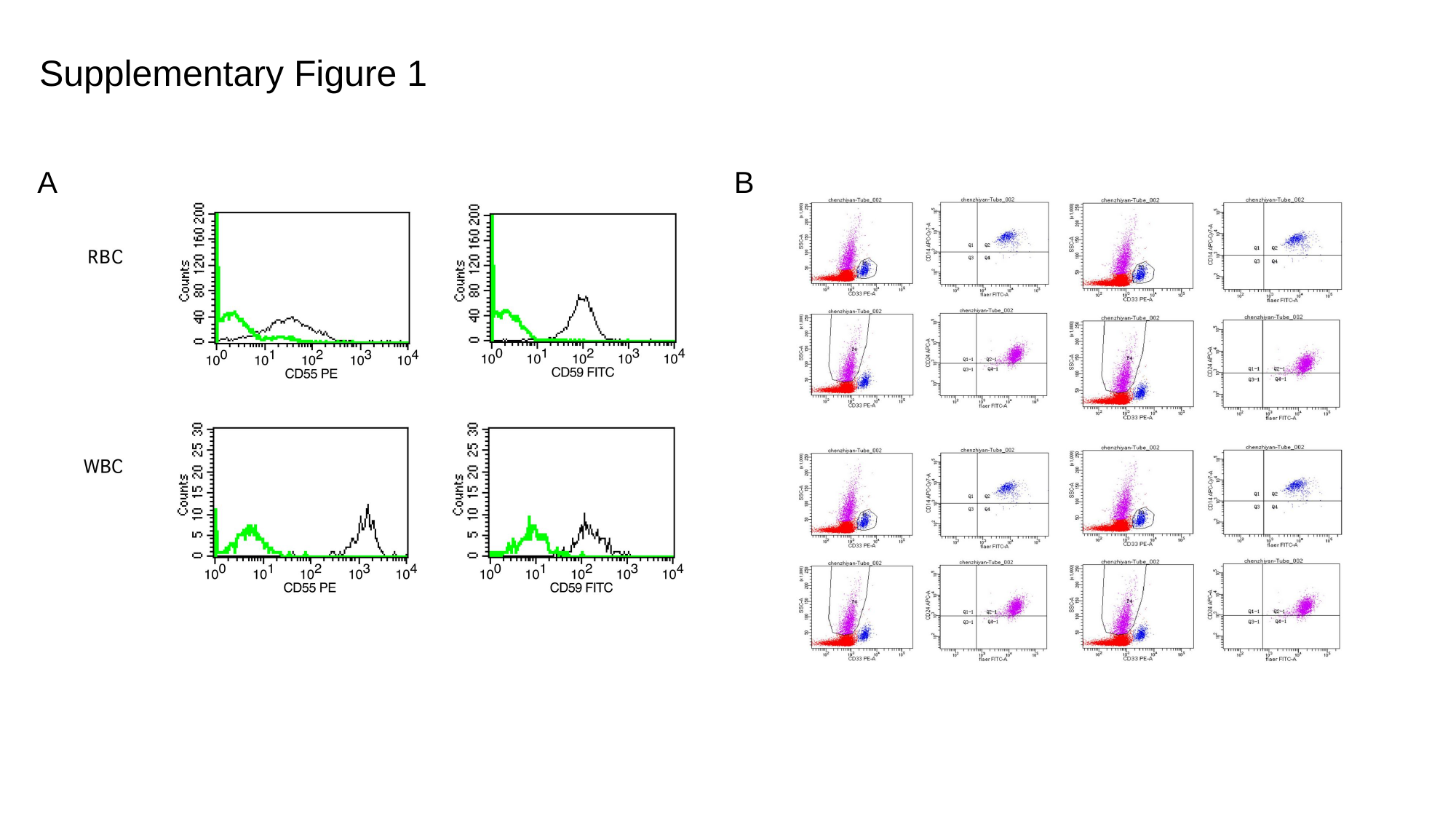

Supplementary Figure 1
A
B

## Slide 2
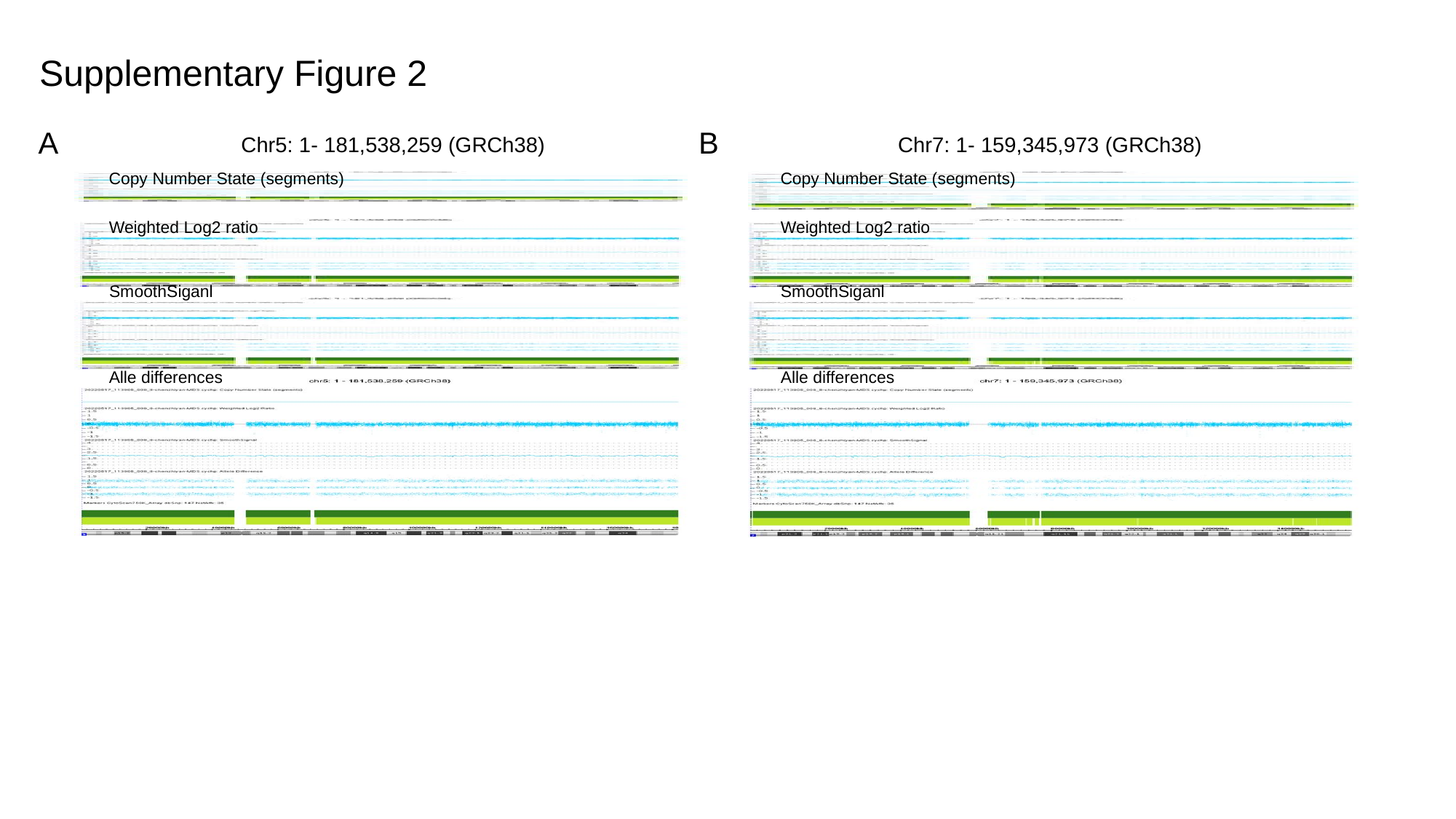

Supplementary Figure 2
A
B
Chr5: 1- 181,538,259 (GRCh38)
Chr7: 1- 159,345,973 (GRCh38)
Copy Number State (segments)
Weighted Log2 ratio
SmoothSiganl
Alle differences
Copy Number State (segments)
Weighted Log2 ratio
SmoothSiganl
Alle differences
